# On a Case of Abscess of the Thoracic Walls, Simulating Pleuritis and Empyema

**Published:** 1848-09

**Authors:** Henry Crisp

**Affiliations:** London


*On a Case of Abscess of the Thoracic Walls, simulating Pleuritis and Empyema.* By HENRY CRISP, M. B., London.—The following case occurred in the practice of my friend Mr. Martin, of Haverhill, Suffolk, who kindly gave me an opportunity of watching the patient throughout. It is interesting, from its similitude to an attack of pleuritis, under which disease, indeed, the patient was at first supposed to be labouring. It has also some points of analogy with the case reported in the number of the Lancet for July 1st, p. 16, by Dr. Macdonnell, of Montreal.

On the first appearance of the swelling it might have been supposed that there was a collection of pus in the pleura, which was making its way externally; but there were no stethoscopic signs which would indicate the presence of empyema.

J. S——, twenty-two years of age, cabinet-maker, of rather intemperate habits, was attacked, in February, 1848, with pain of a sharp, stabbing character in the right side; he was of unhealthy appearance, and one or two members of his family had died of phthisis. The pain was aggravated by making pressure over and between the ribs, by taking a full inspiration, by coughing, and by any motion of the affected side. There was no audible "rubbing sound," nor dulness on percussion. Large crepitation could be heard on both sides of the chest, and the respiratory murmur was present in all parts of the lungs, though very feeble. He had a slight cough, and expectorated a moderate quantity of transparent, viscid mucus; respiration was slightly quickened, and the intercostal muscles of the affected side did not act so freely as those of the other side. These symptoms had been preceded by slight shivering; pulse about 80, small and weak; tongue furred; bowels confined; and he had slight thirst. He was ordered aperient medicines, with small doses of tartar-emetic every five hours, and a mild mercurial at night. This treatment was continued for some time, the patient occasionally feeling better, but never entirely losing the pain. The cough still continued, but he was not confined to his bed; his tongue was always much furred, and great difficulty was experienced in getting his bowels to act properly. His pulse was, for the most part, quicker than natural; his appetite was variable, but generally good.

About three weeks from the appearance of these symptoms, he first noticed a swelling over the site of the pain, just below the right mamma; this was boggy and diffused—not very painful on pressure. At first there was little redness, but after a short time the integuments became of a pinkish hue. There was no dulness of the affected side; the respiratory murmur, though very faint on both sides, was audible all over the chest; pulse about 90, weak; bowels constipated—motions very dark-coloured; his cough still troubles him, and much increases the pain in the side.

About a week after this he had rigors; the swelling became softer, and matter was detected; though this was at first deep-seated, pressure did not empty the sac. Leeches and hot fomentations were employed when the swelling first made its appearance, and appropriate medicines given internally. He became very low; the pulse was quick, weak, and irritable; his tongue was covered with a thick dark fur, and he lost his appetite; the pain was so severe as to prevent his sleeping. Poultices were applied for a few days, and then the abscess was opened. About a teacupful of white, inodorous pus escaped.

Poultices were continued; ammonia and wine, and afterwards quinine, were given internally.

He was very much relieved by the evacuation of the abscess; he slept better, his appetite returned, and he lost his cough entirely. In two or three days, the wound, which had discharged a little, entirely closed, and he gradually but perfectly recovered his former health.—*Lancet.*

